# Factors Affecting Improved Prenatal Screening: A Narrative Review

**DOI:** 10.5539/gjhs.v8n5p160

**Published:** 2015-09-28

**Authors:** Zohreh Shahhosseini, Hoda Arabi, Azam Salehi, Zeinab Hamzehgardeshi

**Affiliations:** 1 Department of Reproductive Health and Midwifery, Nasibeh Nursing and Midwifery Faculty, Mazandaran University of Medical Sciences, Sari, Iran; 2 Traditional and Complementary Medicine Research Centre, Mazandaran University of Medical Sciences, Sari, Iran; 3 Student Research Committee, Mazandaran University of Medical Sciences, Sari, Iran

**Keywords:** prenatal screening, fetus health, prenatal counseling

## Abstract

**Background:**

Prenatal screening deals with the detection of structural and functional abnormalities in the fetus. Health care providers can minimize unintended pregnancy outcomes by providing proper counseling and performing prenatal screening. The purpose of the present review study is to investigate factors affecting improved prenatal screening.

**Methods:**

The present study is a narrative review searching public databases such as Google Scholar and specialized databases such as Pubmed, Magiran, Scientific Information Database, Elsevier, Ovid and Science Direct as well. Using the keywords “prenatal screening”, “fetus health” and “prenatal counseling”, 70 relevant articles published from 1994 to 2014 were selected. After reviewing the abstracts, the full data from 26 articles were ultimately used for writing the present review study.

**Results:**

Three general themes emerged from reviewing the studies: health care providers’ skills, clients’ characteristics and ethical considerations, which were the main factors affecting improved prenatal screening.

**Conclusion:**

Prenatal screening can be successful if performed by a trained and experienced expert through techniques suitable for the mother’s age. Also simultaneously providing proper counseling and giving a full description of the risks and benefits of the procedures for clients is recommended.

## 1. Introduction

Although it is not easy for couples to decide about prenatal screening due to the chance of termination of their pregnancy, prenatal screening is a wise solution for many couples at the risk of having children with serious genetic disorders or birth defects (García, Timmermans, & van Leeuwen, 2012). Prenatal screening indicates the use of various techniques such as genetic and biochemical testing to provide information to pregnant women about the risks of fetal abnormalities (Farina et al., 2003; García et al., 2012; Vallian Boroujeni, 2013). This issue is closely tied to challenging life questions, such as who should survive and who should not. One of the goals of prenatal screening is for couples to obtain information about their pregnancy and, if necessary, to gain access to the most appropriate technique of screening (Dixon & Burton, 2014; Norton, 2008). Another purpose of prenatal screening is to prevent the birth of children with various disorders. In some cases, prenatal treatment can be used to improve intrauterine conditions (Sharrif Moghaddasi, 2011), and if prenatal treatment is not an option, psychological preparation of the parents and necessary medical preparations can help reduce difficulties occurring in the birth of the newborns suffering from congenital anomalies. Although it’s noticeable families are free to continue their pregnancy if they so desire (Bubb & Matthews, 2004; Nussbaum, McInnes, & Willard, 2007).

Although prenatal screening has improved with the medical and treatment advances in prenatal care and the great progress made in screening methods, due to the lack of great improvements in the practicality of embryonic treatments and the inaccessibility of these treatments in many parts of the world, there still remain only two options: continuing pregnancy and accepting the fetus as is or terminating the pregnancy (Turnpenny & Ellard, 2011). It thus appears essential for health care providers to be familiar with prenatal screening and related counseling techniques. As health care providers play a significant role in communicating information to pregnant women, they should have adequate information about different methods of screening and their proper timing, and should pay particular attention to the counseling aspect of prenatal screening (Dormandy & Marteau, 2004; Martin, Hutton, Spelten, Gitsels-van der Wal, & van Dulmen, 2014).

Due to different socio-demographic factors which associated with the uptake of prenatal screening (Gitsels-van der Wal, Verhoeven, et al., 2014) and given the importance of this protocol in different contexts, the present review study was conducted to evaluate factors affecting improved prenatal screening.

## 2. Methods

The present narrative review was conducted in five main steps: Identifying the research questions; Identifying relevant studies; Selecting studies; Collating and summarizing; Reporting the results.

To search for articles, relevant MeSH (Medical Subject Heading) keywords were first determined (prenatal screening, fetus health and prenatal counseling) and the search was then carried out in public databases such as Google Scholar and in specialized databases such as Pubmed, Magiran, Scientific Information Database, Elsevier, Ovid and Science Direct. The articles most frequently cited by well-known authors were investigated and discussed in the results section of the study.

## 3. Results

Reviewing the articles led to three general themes: (1) health care providers’ skills, (2) clients’ characteristics and (3) ethical considerations.

### 3.1 Health Care Providers’ Skills

#### 3.1.1 Health Care Providers’ Practical Skills

Studies show that the skill level of the health care providers performing screening has tremendous effects in reducing the risks and increasing the success of screening (Anuwutnavin, Chanprapaph, Ruangvutilert, Eammatta, & Tontisirin, 2014; Welch & Poulin, 2003). The results of a review study using data from articles on amniocentesis and chorionic villus sampling showed that poor skills in using prenatal screening equipment and underestimating the real risks of screening have adverse consequences (Mujezinovic & Alfirevic, 2007). Thus it is recommended for health care providers to use screening services only according to their professional competency areas (Ardichvili, Jondle, & Kowske, 2010; Hoseinian, 2005; Kariminejad & Kariminejad, 2009).

#### 3.1.2 Health Care Providers’ Knowledge

The knowledge and verbal skills of health care providers contribute considerably to the quality of prenatal counseling services (Fransen et al., 2009; Smith, Slack, Shaw, & Marteau, 1994). The lack of information of health care providers and in turn the lack of appropriate training programs for clients reduces the quality of prenatal screening counseling. It is therefore recommended for all of the health care providers to be aware of the genetic screening tests available in their health care system and to receive training about the efficiency, the advantages and the disadvantages of each of the screening tests. This information should be available to women prior to pursuing them to engage in any screening tests as well (Cousens, Gaff, Delatycki, & Metcalfe, 2014).

#### 3.1.3 Health Care Providers’ Attitude

Positive attitudes of health care providers toward prenatal screening contribute considerably to the clients’ feelings about the valuableness of diagnostic tests and increasing their information (Garel, Gosme-Seguret, Kaminski, & Cuttini, 2002; Gitsels-van der Wal, Manniën, et al., 2014; Rowe, Fisher, & Quinlivan, 2006). Given that making life and death decisions have ethical and emotional consequences for prenatal screening service providers (Garel et al., 2002), it seems they might need to increase their knowledge on the laws, traditions, customs and beliefs of their respective communities in order to assist their clients in making informed decisions about prenatal screening and the treatment measures that may follow (Ahmed, Atkin, Hewison, & Green, 2006; Martin et al., 2014).

#### 3.1.4 Health Care Providers’ Communication Skills

Appropriate communication between health care providers and clients has a great impact on the counseling process of prenatal screening. Development of communication skills contributed considerably to the transfer of information between the clients and the counselors and ultimately the quality of the screening process (Gill & Cowdery, 2014; Smith et al., 1994).

### 3.2 Clients’ Characteristics

#### 3.2.1 Making Informed Choices

By definition, making an informed choice is about deciding based on sufficient levels of relevant information consistent with the decision-maker’s values. However, studies show that a significant percentage of women make uninformed decisions during their prenatal screening due to their insufficient levels of information, the incompatibility of their values or both (Dormandy, Michie, Hooper, & Marteau, 2006). Sufficient understanding of prenatal screening can be a strengthening factor for making informed choices for women visiting health care centers. In addition, women who make more informed choices report less contradiction at the time of their decision-making (van den Berg, Timmermans, ten Kate, van Vugt, & van der Wal, 2005). Although there are no gold standard about what constitutes a sufficient level of information for women qualified for prenatal screening, some crucial points of consensus include: conditions in which screening is recommended, screening test features and interpretations and explanations about possible test results (van den Berg et al., 2005). The compatibility of values is the second requirement for making informed choices and values outline a person’s beliefs on ideal behaviors. Since attitudes can be considered a reflection of a person’s values, it is necessary to explore the clients’ values and attitudes toward preconception screening (Rowe et al., 2006).

#### 3.2.2 Mother’s Age

The mother’s age is an important factor in the incidence of chromosomal abnormalities, as the risk of chromosomal abnormalities such as Down’s syndrome increases with age. However, due to the higher frequency of pregnancies occurring under the age of 35, most of the syndromes pertain to these mothers. As a result, some researchers claim screening to be essential for all pregnant women (Chitayat, Langlois, & Wilson, 2011; Driggers & Seibert, 2008; Kariminejad & Kariminejad, 2009). Another aspect of the impact of the mother’s age on the success of prenatal screening programs is that older women make more informed choices due to their higher levels of knowledge. Nevertheless, according to some studies, older women are likely to reject prenatal screening tests because their decisions about the acceptance of the techniques proposed in the screening are greatly affected by their compatibility with their values (van den Berg et al., 2005). In this way, it’s suggested that screening tests should be carried out using techniques that are consistent with the mother’s age. If these techniques are used earlier than the expected age, they will have complications such as the loss of the amniotic fluid due to fluid leakage, fetal loss and miscarriage (Bubb & Matthews, 2004; Kariminejad & Kariminejad, 2009; Moore & Bhide, 2009). Other complications include increased birth defects, particularly shortened hands and feet as a result of early chorionic villus sampling (Nussbaum et al., 2007; Sharrif Moghaddasi, 2011; Tongsong et al., 2014).

#### 3.2.3 Level of Education

The mother’s high level of education affects with the consistency of her decisions with her values and ultimately the success of the prenatal screening counseling. This relationship seems to be due to the increased level of information of clients and their ability to make more informed choices (van den Berg et al., 2005).

### 3.3 Ethical Considerations

Observing ethical considerations, such as confidentiality, respecting the clients’ rights and refraining from engaging in close personal and dual relationships with the clients, has a major role in the success of the prenatal screening program.

#### 3.3.1 Confidentiality

Confidentiality serves as the basis for developing trust and building a constructive relationship in any counseling program. If the clients do not trust their counselors, they cannot be expected to freely talk about all their problems. It is therefore recommended for both spouses to attend the counseling sessions together, since if one spouse is absent, it is very unlikely for the one present to be able to convey the conclusions drawn in counseling program without alterations. Furthermore, questions tend to arise in these situations that will remain unanswered (Garel et al., 2002; Golden-Grant, Merritt, & Scott, 2015).

#### 3.3.2 Respecting the Clients’ Rights

Information should be gathered from the clients with their consent and with their acceptance of cooperation in the treatment. It is important to respect clients as people with their own rights. Counselors should treat clients the same way they expect to be treated in case they ever need treatment themselves. One of the best ways for protecting the rights of the clients is to develop measures that help them make informed choices (Hoseinian, 2005).

#### 3.3.3 Refraining from Dual Relationships

People set limitations in their relationships that help preserve their identity as an individual. The counselor–client relationship is a special relationship that has been built by the client for a specific purpose. This relationship is not of equality and the counselor is intentionally or unintentionally in the position of power and influence. He works with clients who are very sensitive and vulnerable and when counseling goes beyond its standard limits, it becomes useless and sometimes harmful for the client (Hoseinian, 2005). Another damaging relationship formed in counseling includes multifold relationships built between the client and the counselor, including family, economic, social and commercial relationships, which could entail misuse of the client (Garel et al., 2002).

## 4. Discussion

Given the lack of a definitive treatment for genetic diseases, prenatal diagnosis is the only way for the control and prevention of these diseases in the community. With the advances made in genetic techniques, prenatal diagnosis is successful for almost all of the genetic diseases with their pathogen gene already detected. Early prenatal diagnosis allows couples to make the right decision for continuing the pregnancy. In addition, with prenatal diagnosis, couples who are carriers of genetic diseases can have the chance of giving birth to healthy children (Nussbaum et al., 2007).

Although ethical issues have arisen in all subjects of medicine, this specific issue in medicine has gained particular importance due to the expansion of medical genetics into the society (Kariminejad & Kariminejad, 2009). So maintaining the confidentiality of screening test results is of particular importance. In addition, given the risks and ethical dilemmas associated with prenatal screening programs, the acceptance or rejection of prenatal screening should be based on informed choices (Gitsels-van der Wal et al., 2014). Screening programs should therefore target the empowerment of individuals so that they can make informed choices. Making informed choices means choosing based on ethical considerations and psychological findings. Through making informed choices, the ethical principle of self-determination is respected and more desirable psychological results will be achieved. Moreover, when pregnant women make informed choices, they experience less contradiction in their decisions and will have greater satisfaction. Prenatal screening and counseling measures should therefore be directed toward increasing the frequency of informed choices (Kohut, Dewey, & Love, 2002; van den Berg et al., 2005).

Although some studies show that recommendations for performing prenatal screening as part of routine prenatal care lead to less informed choices, some others suggest that if providing screening is part of routine care, more informed choices are made. In contrast, pregnant women who receive comprehensive information (through books or brochures and counseling by a midwife and gynecologist) make more informed decisions (Dixon & Burton, 2014; Dormandy et al., 2006).

The dominating culture of different countries and their public awareness and training are among the factors affecting decision-making for prenatal screening. For example, in Japan, genetic screening is performed in less than five percent of pregnant women and often during the second trimester and first trimester screening is still uncommon (Yotsumoto et al., 2012). Cultural differences are also effective in determining people’s reactions to screening programs and consequently facilitating informed choices, ensuring self-determination and obtaining informed consent. In other words, the success of prenatal screening is largely proportional to the level of knowledge, freedom and sense of security in the society, and when these conditions are not provided for the clients, the success and usefulness of the screening results is wishful thinking. Even when prenatal screening tests are strongly recommended, the client should be treated so that this suggestion seems completely arbitrary (Ardichvili et al., 2010; Fransen et al., 2009; Hosseini Bereshneh & Sadr-Nabavi, 2014).

In conclusion, the quality of prenatal screening and the outcomes of using these methods are affected by certain characteristics of the health care providers and clients and also by ethical and counseling issues. Through health care providers’ communication skills, sufficient knowledge and positive attitudes toward making informed choices concerning screening. In this way, they can help empower the clients and enable them to choose according to their values and based on sufficient knowledge and consequently reduce adverse psychological consequences in people who resort to screening for the detection of fetal abnormalities.
